# Activity of a Carbohydrate-Binding Module Therapy, Neumifil, against SARS-CoV-2 Disease in a Hamster Model of Infection

**DOI:** 10.3390/v14050976

**Published:** 2022-05-06

**Authors:** Rachel Fell, Jane A. Potter, Samantha Yuille, Franscisco J. Salguero, Robert Watson, Didier Ngabo, Karen Gooch, Roger Hewson, David Howat, Stuart Dowall

**Affiliations:** 1 United Kingdom Health Security Agency (UKHSA), Porton Down, Salisbury SP4 0JG, UK; rachel.fell@phe.gov.uk (R.F.); javier.salguero@phe.gov.uk (F.J.S.); robert.watson@phe.gov.uk (R.W.); didier.ngabo@phe.gov.uk (D.N.); karen.gooch@phe.gov.uk (K.G.); roger.hewson@phe.gov.uk (R.H.); 2 Pneumagen Ltd., Kinburn Castle, Doubledykes Road, St Andrews, Fife KY16 9DR, UK; jane.potter@pneumagen.com (J.A.P.); samantha.yuille@pneumagen.com (S.Y.); david.howat@pneumagen.com (D.H.)

**Keywords:** COVID-19, SARS-CoV-2, therapy, host targeted

## Abstract

The rapid global spread of severe acute respiratory coronavirus 2 (SARS-CoV-2) has resulted in an urgent effort to find efficacious therapeutics. Broad-spectrum therapies which could be used for other respiratory pathogens confer advantages, as do those based on targeting host cells that are not prone to the development of resistance by the pathogen. We tested an intranasally delivered carbohydrate-binding module (CBM) therapy, termed Neumifil, which is based on a CBM that has previously been shown to offer protection against the influenza virus through the binding of sialic acid receptors. Using the recognised hamster model of SARS-CoV-2 infection, we demonstrate that Neumifil significantly reduces clinical disease severity and pathological changes in the nasal cavity. Furthermore, we demonstrate Neumifil binding to the human angiotensin-converting enzyme 2 (ACE2) receptor and spike protein of SARS-CoV-2. This is the first report describing the testing of this type of broad-spectrum antiviral therapy in vivo and provides evidence for the advancement of Neumifil in further preclinical and clinical studies.

## 1. Introduction

Coronaviruses (CoVs) are roughly spherical enveloped RNA viruses that belong to the *Coronaviridae* family under the order *Nidovirales* [1]. To date, seven coronavirus strains have been identified which have crossed the species barrier to infect humans. Four of these are endemic human coronavirus (HCoV) strains that generally cause a mild common cold illness—229E, NL63, OC43, and HKU1 [2]. The remaining three novel strains have caused serious outbreaks in the 21st century. The first, termed severe acute respiratory syndrome (SARS)-CoV, started in the Guangdong Province of southern China, before spreading to 29 countries, affecting over 8000 people and causing 774 deaths [3]. The second, Middle East respiratory syndrome (MERS)-CoV, is causing an ongoing outbreak in the Arabian Peninsula which started in 2012. Between April 2012 and December 2019, there were 2499 confirmed cases and 858 deaths [4]. The third, SARS-CoV-2, was identified following reports of several patients experiencing atypical pneumonia in Wuhan city, China, in December 2019 [5]. Despite massive efforts to control the transmission, the virus rapidly spread globally [6].

SARS-CoV-2 infection is initiated by the recognition and binding of the angiotensin-converting enzyme 2 (ACE2) on the host cell surface by the SARS-CoV-2 spike (S) protein [7]. The cellular transmembrane protease serine 2 (TMPRSS2) is recruited to cleave the viral S protein enabling membrane fusion [8]. Novel therapeutic approaches are urgently needed: the blocking of the S protein interaction with the ACE2 receptor and the deactivation of processing via TMPRSS2 have been identified as potential strategies to prevent viral cell entry [9].

**Figure 1:**
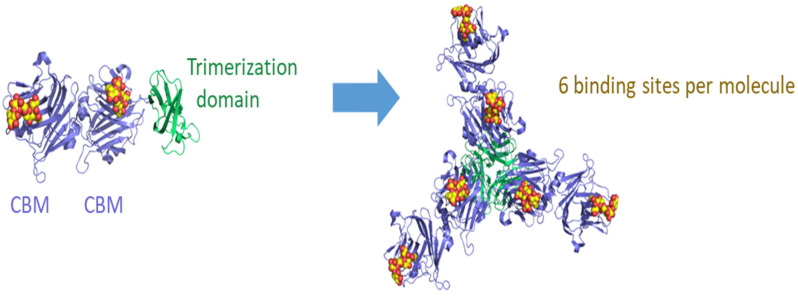
Schematic diagram of CBM domain arrangement and trimerisation in the Neumifil compound. Bound 2,3 sialyllactose molecules (shown as spheres) indicate the ligand-binding sites. The image was produced with PyMOL Molecular Graphics System (Version 2.0., Schrödinger, LLC). PDB accession codes: 4c1w and 2w38.

The targeting of cellular factors essential for virus infection and replication provides an innovative approach for antiviral therapy, as host proteins are less likely to acquire resistance than viral proteins [10]. One such approach uses engineered carbohydrate-binding modules (CBMs) isolated from bacteria, such as Vibrio cholerae [11] and Streptococcus pneumoniae [12]. CBMs mask cellular receptors through high-affinity binding to glycans, preventing viral attachment [12]. One such sialic acid-binding CBM (*Sp*2CBMTD), from the family 40 domain of S. pneumoniae neuraminidase A sialidase, is formed by linking three CBM dimers to a trimerisation domain, derived from Pseudomonas aeruginosa pseudaminidase, resulting in six binding sites per molecule (Figure 1). *Sp*2CBMTD has been demonstrated to confer protection in mice from a lethal influenza virus challenge with several strains: H7N9, H5N1 [10], and H1N1 [12]. Whilst ACE2 binding is well recognised as a key interaction for SARS-CoV-2, molecular modelling also predicts an interaction of the S protein with sialic acids [13,14]. This suggests that SARS-CoV-2 may bind to sialic acid regions exposed to the cell membrane and favour the subsequent interaction between the S protein and ACE2 [15]. This hypothesis is supported by in vitro studies which have demonstrated binding of the S protein to cells with low levels of ACE2 [16], suggesting a complex interaction with multiple cell-surface receptors, such as sialylated glycans, may facilitate SARS-CoV-2 binding to host cells [17]. Therefore, the testing of a broad-spectrum antiviral known to bind sialic acid for activity against SARS-CoV-2 infection and disease progression is warranted.

Multivalent forms of CBM (family 40 domain) from *S. pneumoniae* have been engineered to ensure high-affinity adherence to sialic acid receptors in the respiratory tract [18]. Neumifil, a first-in-class CBM, is derived from *Sp*2CBMTD, with modifications that reduce its predicted immunogenicity in humans while retaining its ligand binding specificity and affinity. Neumifil is being developed as a self-administered intranasal spray by Pneumagen Ltd. to primarily target the nasal lining and provide a defence against respiratory pathogen entry.

## 2. Materials and Methods

### 2.1. Ethical Statement

All experimental protocols with animals were undertaken according to the United Kingdom Animals (Scientific Procedures) Act 1986, with studies conducted under the authority of a UK Home Office approved project licence. The experimental protocols were approved by ethical review at Public Health England (PHE) by the Animal Welfare and Ethical Review Body (AWERB) on 15 July 2021 (Approval Code: PPL PDC57C033). This research is reported in accordance with the ARRIVE guidelines (https://arriveguidelines.org, accessed on 1 March 2022). Prior to the start of the study, humane clinical endpoints were set which consisted of 20 % weight loss, compared with baseline; inactivity/immobility; neurological signs; or on the advice of severe disease from the Named Animal Care and Welfare Officer (NACWO).

### 2.2. Animals

In total, 15 Golden Syrian hamsters, aged 5–6 weeks at arrival, were obtained from a UK Home Office accredited supplier (Envigo, UK). Animals were randomly assigned to one of three groups: naïve control (*n* = 3 males); mock-treated with vehicle (*n* = 3 males and *n* = 3 females); and Neumifil pre-treated (*n* = 3 males and *n* = 3 females). Six animals per treatment group were used based on a one-way analysis of variance (ANOVA) calculation to compare viral titres, with a 1 log difference, power at 80%, and a significance level of 0.05. All animals were evaluated for signs of ill health on arrival and were approved for release in the study by the Named Animal Care and Welfare Officer (NACWO). Animals were singly housed during the study since SARS-CoV-2 is known to be highly transmissible by close contact [19,20]. Access to food and water was ad libitum, and environmental enrichment was provided.

### 2.3. Neumifil Pre-Treatment

Neumifil (9.4 mg/mL) and vehicle (comprised of Neumifil formulation buffer) were provided by Pneumagen and stored at −60 °C before use. Neumifil was prepared using a proprietary *E. coli* expression system by a contract manufacturing organisation. On each dosing day, aliquots were defrosted immediately prior to delivery. Animals were dosed by administering 100 µL treatments intranasally (50 µL per nare) at −7, −3, and −1 days pre-challenge.

### 2.4. Virus Challenge

The virus used in this study was SARS-CoV-2 Victoria/01/2020 [21], generously provided by The Peter Doherty Institute for Infection and Immunity, Melbourne, Australia, as passage 1, and it was further passaged twice in Vero/hSLAM cells (European Collection of Cell Cultures, Salisbury, UK). The virus was diluted in PBS to intranasal instillation, in a volume of 100 µL per nare, and given under isoflurane sedation. Back-titration confirmed that the final challenge dose was 1554 pfu.

### 2.5. Clinical Observations

Throughout the study, clinical signs were recorded at the two extremes of the working day (07:00–10:00 and 14:00–16:00) by experienced husbandry and animal welfare staff. At the earlier time point, animals were weighed. Clinical signs of disease were assigned a score based on the following criteria: 0, healthy; 2, ruffled fur, wet tail, dehydrated; 3, wasp-waisted, arched back, eyes closed; and 5, laboured breathing. A cumulative score to combine all observed signs was then assigned for each animal at that time point. This scoring system is based on previous studies with the hamster COVID-19 model [22] and other viral disease models, including Ebola [23], Zika [24], Lassa [25], and Crimean–Congo haemorrhagic fever viruses [26].

### 2.6. Necropsy Procedures

Hamsters were anaesthetised with isoflurane and then given an overdose of sodium pentobarbitone. A necropsy was performed immediately after confirmation of death. Nasal washes, throat swabs, and lung samples were collected and stored at −80 °C for virology analysis. Lung samples were collected directly into RNAlater solution (QIAGEN, Hilden, UK) for RNA analysis or a dry tube for live virus assays. The remainder of the lungs and the head (for sampling the nasal cavity) were collected into histology pots containing 10% neutral-buffered formalin.

### 2.7. Sample Preparation

Lung tissue samples were weighed and then added to reinforced PreCellys tubes containing ceramic beads (Stretton Scientific, Alfreton, UK). Samples for RNA extraction were homogenised with RLT buffer supplemented with 1% (*v*/*v*) beta-mercaptoethanol (QIAGEN, UK) using an automated PreCellys21 homogeniser (Stretton Scientific, UK). Samples for the focus-forming unit assay were homogenised with PBS. Non-tissue samples for RNA extraction were inactivated in AVL buffer (QIAGEN) and ethanol. Downstream RNA extraction on all samples was performed using the BioSprint One-For-All Vet Kit (Indical, UK) and the Kingfisher Flex Platform (Thermo-Fisher, UK) as per the manufacturers’ instructions.

### 2.8. Quantification of Viral Loads by RT-qPCR

Reverse transcription-quantitative polymerase chain reaction (RT-qPCR) targeting a region of the SARS-CoV-2 nucleocapsid (N) gene was used to determine viral loads and was performed using TaqPath™ 1-Step RT-qPCR Master Mix, CG (Applied Biosystems™), 2019-nCoV CDC RUO Kit (Integrated DNA Technologies, Coralville, IA, USA) and QuantStudio™ 7 Flex Real-Time PCR System. Sequences of the N1 primers and probe were as follows: 2019-nCoV_N1-forward, 5′ GACCCCAAAATCAGCGAAAT 3′; 2019-nCoV_N1-reverse, 5′ TCTGGTTACTGCCAGTTGAATCTG 3′; 2019-nCoV_N1-probe, 5′ FAM-ACCCCGCATTACGTTTGGTGGACC-BHQ1 3′. The cycling conditions were as follows: 25 °C for 2 min, 50 °C for 15 min, 95 °C for 2 min, followed by 45 cycles of 95 °C for 3 s, 55 °C for 30 s. The quantification standard was in vitro transcribed RNA of the SARS-CoV-2 N ORF (Accession Number NC_045512.2), with quantification between 1 × 10^1^ and 1 × 10^6^ copies/µL.

### 2.9. Foci-Forming Assay (FFA)

Fluid samples and lung tissue homogenate were serially diluted before being added, in duplicate, to Vero E6 cell monolayers in 96-well flat-bottomed culture plates (seeded 24 h before) for 1 h at 37 °C. Samples were removed, overlay media was added, and then the plates were incubated for 24 h at 37 °C. Plates were fixed overnight by adding 40% formalin and fumigating. The following day, plates were washed with water and incubated with 0.3 % hydrogen peroxide for 20 min at room temperature. The plates were washed again with sterile PBS and incubated with an anti-SARS-CoV-2 primary antibody for 1 h at room temperature and then washed and incubated for a further hour with a secondary goat anti-rabbit IgG HRP conjugate. After a further PBS wash, the plates were incubated for 10 min with TrueBlue substrate, washed with water, and left to dry for a minimum of 3 h, before counting using the CTL scanner.

### 2.10. Histopathological Analysis

Samples from the left cranial and left caudal lung lobes and the nasal cavity were fixed by immersion in 10% neutral-buffered formalin and processed routinely into paraffin wax. Nasal cavity samples were decalcified using an EDTA-based solution prior to embedding. Then, 4 µm sections were cut and stained with haematoxylin and eosin (H&E) and examined microscopically. In addition, samples were stained using the RNAscope technique to identify SARS-CoV-2 viral RNA. Briefly, tissues were pre-treated with hydrogen peroxide for 10 min (room temperature), target retrieval for 15 min (98–101 °C), and protease plus for 30 min (40 °C) (Advanced Cell Diagnostics, Newark, CA, USA). A V-nCoV2019-S probe (Cat No. 848561, Advanced Cell Diagnostics) was incubated with the tissues for 2 h at 40 °C. Amplification of the signal was carried out following the RNAscope protocol using the RNAscope 2.5 HD Detection Kit—Red (Advanced Cell Diagnostics). The slides were scanned digitally using a Hamamatsu S360 digital slide scanner and examined using ndp.view2 software (v2.8.24). Nikon NIS-Ar software was used to perform digital image analysis in order to calculate the area of pneumonia and quantify the presence of viral RNA in lung sections. Digital image analysis was carried out in order to calculate the total area of the lung section that was positive for viral RNA.

A semiquantitative scoring system developed by an experienced team of veterinary pathologists in collaboration with international groups working on the SARS-CoV-2 hamster model was applied to evaluate the severity of lesions in the lung and nasal cavity. For the nasal cavity, a semiquantitative scoring system was applied to evaluate the presence of virus RNA: 0 = no positive staining; 1 = minimal; 2 = mild; 3 = moderate; and 4 = abundant staining. These scoring systems are described in greater detail elsewhere [22].

All slides were digitally scanned, and evaluation was performed by qualified veterinary pathologists blinded to the individual and treatment groups.

### 2.11. Neumifil-Binding ELISAs

SARS-CoV-2 spike-Neumifil binding ELISA: The spike proteins (Stratech, Ely, UK) were recombinantly expressed in HEK293 cells and encompassed either the S1 segment (residues 1–685) or the receptor-binding domain (RBD), residues 306–527 of several SARS-CoV-2 variants. Binding interactions between Neumifil and recombinant spike proteins were measured using a standard ELISA. Spike S1 or RBD proteins were coated (0.1 µg per well) onto 96-well high binding plates and incubated overnight at 4 °C. The plates were washed with PBS containing 0.05 % (*v*/*v*) Tween-20 and incubated with a dilution series of Neumifil (0 to 29.2 µg/mL for S1 coated plates or 0 to 41.0 µg/mL for RBD plates), in triplicate. Immunodetection of Neumifil binding was performed by incubation with polyclonal rabbit anti-Neumifil, followed by HRP-labelled goat anti-rabbit IgG and TMB substrate development. Absorbance was measured at 450 nm (620 nm values were used as reference). For each ligand, an EC_50_ value (corresponding to the Neumifil concentration that gave half-maximal binding) was calculated from a 4-parameter logistic (4PL) regression fit of the dose–response curve.

Human ACE2-Neumifil binding ELISA: A similar ELISA method was used to measure the hACE2–Neumifil interaction: recombinant hACE2 was coated onto plates (0.1 µg/well), and the protocol for detection of Neumifil binding (0 to 29.2 µg/mL) was followed as described above.

### 2.12. Statistical Analysis

Statistical analyses were performed using Minitab, version 16.2.2 (Minitab Inc., State College, PA, USA). A non-parametric Mann–Whitney statistical test was applied to ascertain significance between groups. A significance level below 0.05 was considered statistically significant.

## 3. Results

### 3.1. Effect of Neumifil Pre-Treatment on Clinical Disease Progression

**Figure 2:**
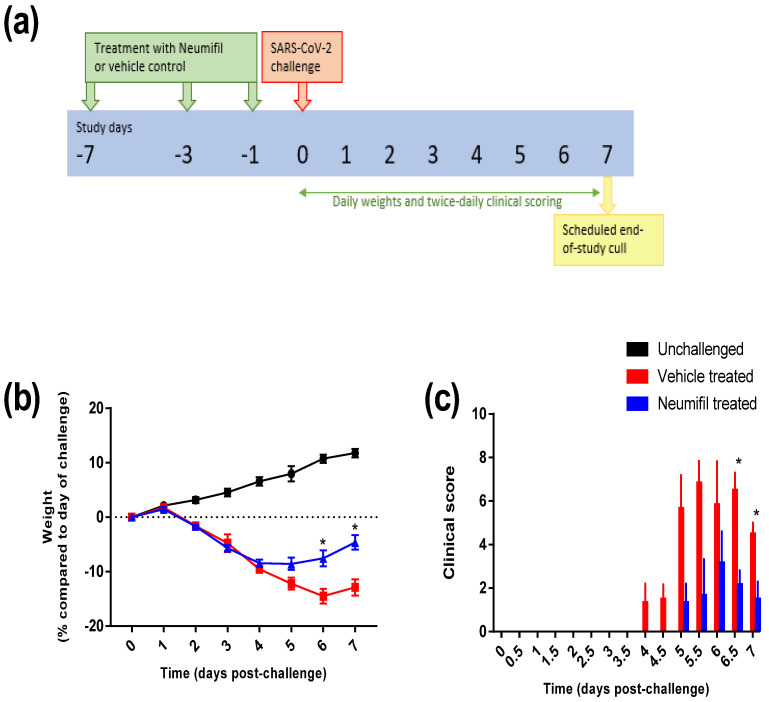
Clinical disease progression of Neumifil-treated compared with mock-treated hamsters after challenge with SARS-CoV-2: (**a**) schematic overview of the study, with hamsters receiving Neumifil three times prior to challenge; (**b**) changes in weight compared with the day of challenge; (**c**) clinical scores. Data show mean values with error bars denoting standard error of the mean. *, *p* < 0.05 for comparison between vehicle-treated and Neumifil-treated groups (Mann–Whitney test). *n* = 6 hamsters per group.

Groups of six hamsters were intranasally dosed with Neumifil or the vehicle control on three occasions (−7, −3, and −1 days pre-challenge) in the week preceding the SARS-CoV-2 challenge (Figure 2a). After the challenge, all animals exhibited weight loss, whereas the naïve control animals housed alongside continued to gain weight (Figure 2b). However, on day 5 following the viral challenge, animals in the Neumifil-treated group started to regain weight; the results were statistically significant, compared with untreated animals on days 6 and 7 (*p* = 0.0104 and *p* = 0.0202, respectively). Clinical signs in untreated animals started on day 4 post-challenge, whereas for Neumifil-treated animals, they were first recorded after 5 days (Figure 2c). Scores in the Neumifil-treated groups were consistently lower than those for the mock-treated groups, approaching significance on days 5 and 5.5 post-challenge (*p* = 0.0656 and *p* = 0.0547, respectively) but reaching the threshold on days 6.5 and 7 (*p* = 0.0104 and *p* = 0.0374, respectively).

### 3.2. Effect of Neumifil Pre-Treatment on Viral Load

**Figure 3:**
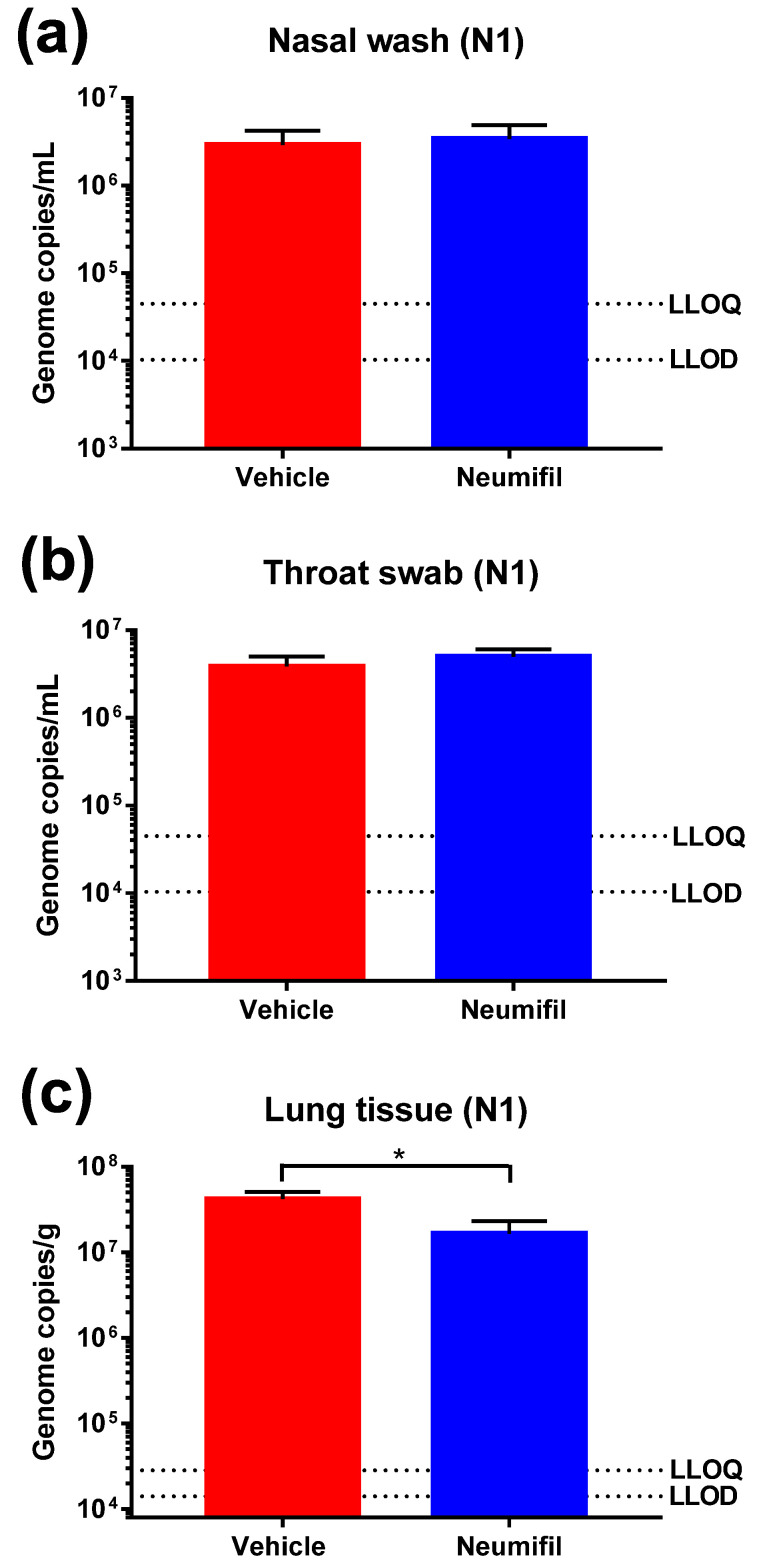
Viral RNA levels in (**a**) nasal washes, (**b**) throat swabs, and (**c**) lung tissue samples taken from Neumifil- and mock-treated hamsters 7 days after SARS-CoV-2 challenge. Samples were tested in an N1-based PCR assay. LLOQ, lower limit of quantification; LLOD, lower limit of detection. *, *p* < 0.05 (Mann–Whitney test). *n* = 6 hamsters per group.

Nasal washes, throat swabs, and lung samples were tested for viral load at the end of the study (7 days post-challenge) (Figure 3). Viral RNA was detected using the N1-based assay, and a significant difference was observed in the lung of the Neumifil-treated animals, compared with the untreated controls (*p* = 0.0453) (Figure 3c). No live virus was detected in any of the samples tested (data not shown).

### 3.3. Histological Changes in Neumifil-Treated Hamsters Compared with Mock-Treated Animals

Lesions consistent with infection with SARS-CoV-2 [22,27] were observed in the lungs and nasal cavities of all animals challenged with SARS-CoV-2. In contrast, only minimal, non-specific inflammatory changes were noted in the naïve control group.

**Figure 4:**
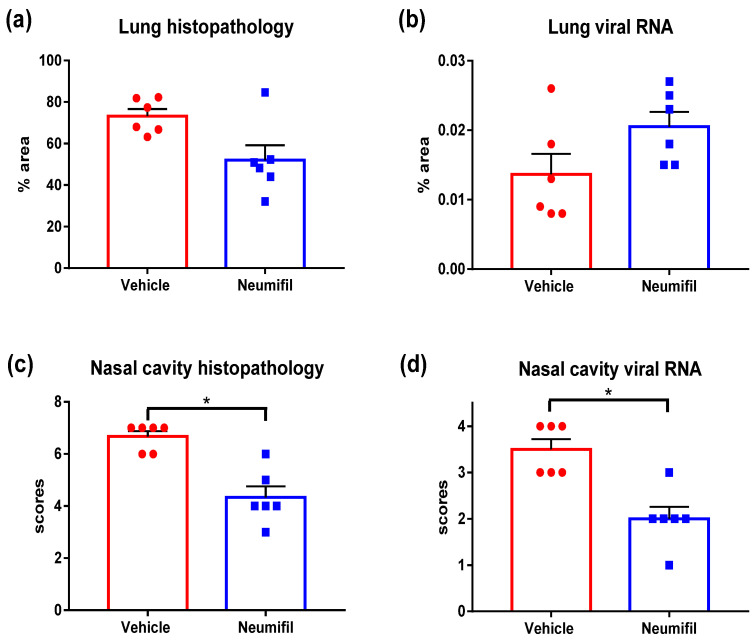
Quantitative analysis of histological specimens from Neumifil-treated and vehicle-treated animals after SARS-CoV-2 challenge: (**a**) percentage of area of pneumonia in the lung; (**b**) percentage of area positively stained for viral RNA in the lung using in situ hybridisation; (**c**) cumulative scores of nasal cavity lesions (presence of exudates and necrosis of epithelium); (**d**) subjective scores of viral RNA presence in the nasal cavity using in situ hybridisation. Bars show mean values with error bars denoting standard error of the mean. * *p* < 0.05 (Mann–Whitney test). *n* = 6 hamsters per group.

Disease severity in the lung, characterised by both percentage of pneumonic change and individual severity of histopathological changes associated with SARS-CoV-2 infection, was greatest in the vehicle-treated group. By comparison, the data showed a reduction in severity in the animals which received Neumifil pre-treatment (Figure 4a). The amount of viral RNA detected in the lung in both groups appeared similar (Figure 4b). None of the changes in the lung were statistically significant.

**Figure 5:**
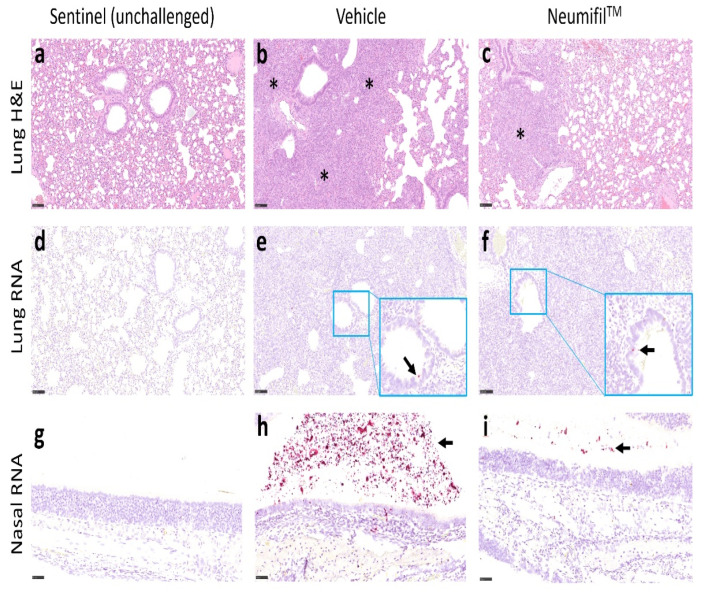
Representative images of lung histopathology (H&E) and presence of viral RNA (in situ hybridisation) in respiratory samples. (**a**–**c**) Lung histolopathology. Areas of bronchopneumonia (*) were observed in the lungs from both vehicle-treated and Neumifil-treated animals, with a lower severity in the Neumifil-treated group. (**d**–**f**) Presence of viral RNA in the lung. A small amount of virus RNA was detected in the airway epithelium and inflammatory infiltrates within the lung and olfactory/respiratory epithelium (arrows). (**g**–**i**) Presence of viral RNA in the nasal cavity. Inflammatory infiltrates and exudates were detected within the nasal cavity (arrows). Scale bars on lung images represent 100 μm and on nasal cavity images represent 50 μm.

The lung lesions consisted of a broncho-interstitial pneumonia with areas of consolidation. Large numbers of inflammatory cells, primarily macrophages, and neutrophils, with some lymphocytes and plasma cells, infiltrated alveolar walls and filled alveolar spaces; prominent type II alveolar hyperplasia was noted in some areas, alongside alveolar oedema. The airways were also infiltrated by similar inflammatory cells. Whilst larger airways contained fewer inflammatory cells, the changes in smaller airways were of increased severity, with concomitant epithelial degeneration and loss. Vessels were variably surrounded by lymphocytes (perivascular cuffing), occasionally infiltrating the walls. The severity of these changes differed between the animals in each group (Figure 5a−c). Cells positive for viral RNA were sparsely dispersed, with positive cells tending to be on the outskirts of alveoli (Figure 5d−f).

In the nasal cavity, data demonstrated a significant reduction in the severity of histopathological lesions in the Neumifil-treated group (*p* = 0.0082) (Figure 4c). Similarly, there was a significant reduction in viral RNA staining of the nasal cavity in Neumifil-treated animals compared with the vehicle-treated group (*p* = 0.0104) (Figure 4d).

The lesions in the nasal cavity were characterised by the presence of exudates (fluid with inflammatory cells, mainly neutrophils but also mononuclear cells) and necrosis of the epithelium in the respiratory and olfactory mucosae. Cells staining for viral RNA tended to be focussed on the exposed surface of the nasal cavity, with a noticeable decrease in staining in the group treated with Neumifil (Figure 5g−i).

### 3.4. Neumifil Binding ELISAs

**Figure 6:**
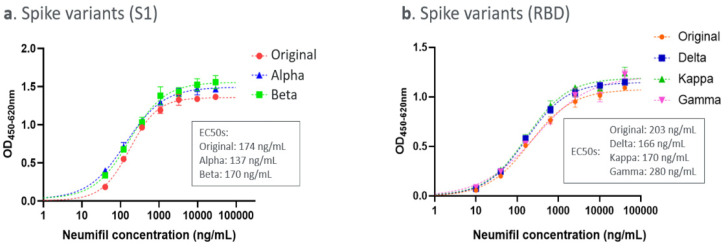
Detection of Neumifil binding to SARS-CoV-2 Spike proteins tested by ELISA technique: (**a**) spike S1 and (**b**) spike RBD. The dotted lines represent 4PL curve fits of the data. Inset: EC50 values for each variant.

**Figure 7:**
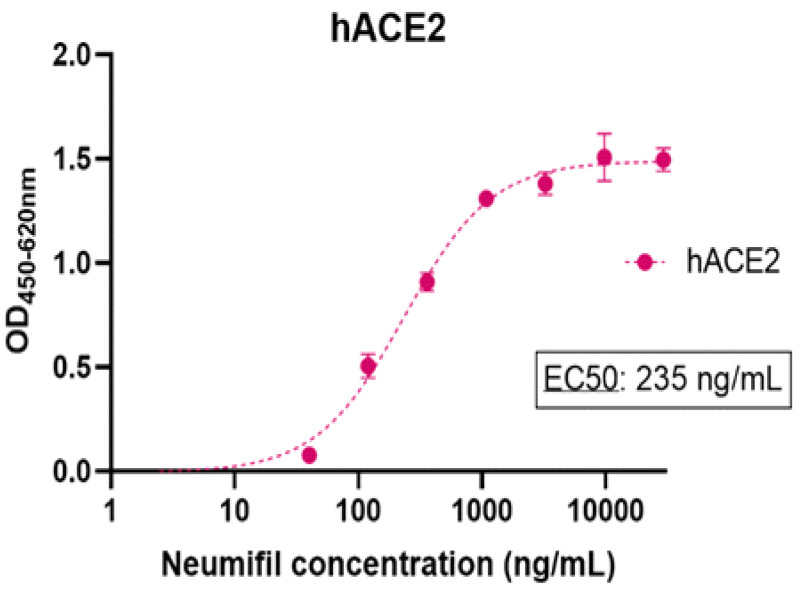
Detection of Neumifil binding to recombinant human ACE2 tested by ELISA technique. The dotted line represents a 4PL curve fitting of the data. Inset: EC50 value.

As shown in Figure 6a, Neumifil binds to the original Spike S1 protein with a half-maximal effective concentration (EC50) of 174 ng/mL. The binding profiles and EC50s for the variants indicate that the affinity is not significantly affected by the mutations present in the Alpha and Beta variant sequences. Similarly, there is no significant difference in the Neumifil-binding profile of the Delta, Kappa, or Gamma RBD proteins, compared with the unmutated RBD (Figure 6b). Neumifil also directly binds to hACE2 with an EC50 for the interaction of 235 ng/mL (Figure 7). No binding of Neumifil to negative control wells of bovine serum albumin was observed (data not shown).

## 4. Discussion

The SARS-CoV-2 hamster model used in this report recapitulates many of the human disease characteristics and human tissue tropism, which minimises complications in interpretation between species [28]. The World Health Organisation COVID-19 modelling (WHO-COM) expert working group has noted the benefits of this animal model and that work in hamsters can be completed quickly and in a cost-effective manner [29].

Glycan screening arrays have demonstrated that *Sp*CBM (parent molecule of the active component of Neumifil) recognises both α2,3- and α2,6-linked sialic acids [11] and, thus, is able to bind throughout the respiratory tract in humans. The distribution of sialic acid receptors in hamsters is similar to that found in ferrets, another widely used model for respiratory pathogen research such as that on influenza virus [30], and closely resembles human sialic acid distribution. In humans, both α2,3- and α2,6-linked sialic acids are expressed in the upper respiratory tract, while the former is predominant in the lungs [31]. Therefore, differences in the relevant physiology between the preclinical hamster animal model and humans are minimal.

In the current study, hamsters were given 940 μg Neumifil intranasally on days 7, 3, and 1 pre-challenge, in line with doses used for similar murine CBM studies in which doses of 50–500 μg showed effects [12]. A substantial toxicity package was already available for Neumifil in preparation for the commencement of human clinical studies (following testing in non-human primates and rats), and no observed side effects were found in mice. Thus, a group consisting of just Neumifil delivery was not warranted in this study, so reducing the number of animals used. After challenge with SARS-CoV-2, animals pre-treated with Neumifil still showed signs of disease but demonstrated less weight loss, faster weight recovery, and lower clinical scores than those receiving vehicle treatment alone. The weight loss observed in our study is comparable to that reported by others [32,33], despite the fact that our viral challenge dose was up to two logs lower than that used previously. Indeed, we have reported weight loss with doses as low as 16 pfu [22]. Due to these variations between different groups and challenge doses, and the fact that hamsters are outbred and housed in non-uniform conditions, the incorporation of the vehicle-only group was essential to control data from the treated group.

The results reported here with SARS-CoV-2 are similar to those reported in a CBM study with influenza virus, in which expedited weight loss recovery was observed in CBM-treated animals [12]. The clearance of the virus by the immune system may confer protection against subsequent exposure to the virus, thus extending the benefits; this was previously observed in an influenza model with *Sp*CBM treatment in which sufficient antibodies were generated to protect against a secondary challenge [10]. Other therapies evaluated in vivo against SARS-CoV-2 in the hamster model have also shown milder disease and less weight loss in treated groups [34,35,36]. Although CBM treatment has been shown previously to stimulate cytokine production in a mouse model of influenza virus [10], this was not explored in the current study, as immunological reagents for hamsters are not widely available at the current time.

No viable virus was detected in any of the samples collected at necropsy 7 days post-challenge, but this was expected given that it is reported that live virus is cleared by this time point [19]. As Neumifil is expected to prevent entry at the cell surface, the collection of sequential samples such as nasal washes and throat swabs was avoided, as these procedures would run the risk of removing the compound and negating its potential effect. Therefore, the study was designed to characterise the course of disease progression and assessment of local damage caused by the infection. Although Neumifil was delivered intranasally, a significant decrease in viral RNA present in the lungs was observed using the N1 assay (which detects viral remnants), suggesting that the viral burden during infection was reduced by the Neumifil pre-treatment. In mice, it has been demonstrated that *Sp*2CBMTD (Neumifil parent CBM) is detected in the lungs for up to 7 days after a single intranasal administration [12].

The pathology results showed no significant differences in lung viral nucleic acid levels between Neumifil-treated and vehicle-treated animals. This was in contrast to the results from the PCR assay which showed significantly less viral RNA in Neumifil-treated animals. There were significant differences in the pathology results for the nasal cavity, with lower lesion and RNA scores in animals treated with Neumifil. Given the intranasal delivery of the compound, it is expected that the concentration of Neumifil in the upper respiratory passages would be high which may explain this effect—although PCR results from the nasal cavity showed no differences. These disparities between the two assays may be due to the PCR assay being a quantitative output from a larger volume of tissue, whereas the RNAscope is qualitatively based on a more refined sample area. In addition, the PCR readout is based on the detection of nucleoprotein, whereas the RNAscope is directed to the spike protein sequence. Nonetheless, these results warrant further investigation with future studies to investigate optimal dosing strategies and dissemination of Neumifil across the surface of the whole hamster respiratory tract. Whilst some of the changes in viral load met significance thresholds using the PCR and in situ hybridisation methods, the reduction in actual levels was small, compared with the effects on clinical outcomes. Our hypothesis is that the Neumifil acts by inhibiting initial virus entry from the challenge virus, but also by inhibiting entry by subsequent virus generations shed by infected cells. Therefore, whilst Neumifil might not have prevented all entry of SARS-CoV-2 virions, it may have contributed to reducing local spread, thus resulting in a positive clinical outcome. This is further corroborated by weight loss being similar 4 days post-challenge due to the initial infection but then resolving thereafter in the animals that received Neumifil, coupled with lower clinical scores in the treated group. In the Syrian hamster model of SARS-CoV-2 infection, the weight loss profile over the first 7 days of infection correlates more closely with markers of inflammation than viral titre levels [32].

The SARS-CoV-2 spike protein is highly glycosylated, with 22 predicted N-linked glycosylation sites and 3 O-linked glycosylation sites [37]. ELISA experiments were performed to determine whether or not Neumifil interacts with the SARS-CoV-2 spike protein and whether any such interaction is affected by new variant mutations. ELISAs demonstrated high-affinity binding of Neumifil to both S1 and RBD forms of the spike protein, with EC_50_ values of 174 ng/mL and 203 ng/mL, respectively (corresponding to 1.1 and 1.3 nM Neumifil). Moreover, SARS-CoV-2 spike proteins representing the Alpha, Beta, Gamma, Delta, and Kappa variants were recognised by Neumifil, with similar high affinities, indicating that the mutations present in these variants of concern do not affect the Neumifil–spike interaction. For consistency with international studies, a widely distributed virus strain that was isolated early in the pandemic was used for challenge studies [21]. These studies could, in the future, be conducted with variant strains as they become available. Neumifil also binds to hACE2, confirming its potential to target both viral and host sialic acid.

Due to viral strain heterogeneity and the inability to predict the future, broadly active therapeutics which target conserved pathways should be championed [28]. Given that variants of SARS-CoV-2 can breakthrough vaccine-induced immunity [38,39], it is essential to increase the toolbox of interventions against COVID-19. Here, we showed that Neumifil reduces symptoms and shortens the clinical manifestation of SARS-CoV-2 disease in a stringent preclinical model. The intention is to use Neumifil in subject pools at a high risk of exposure prior to infection, as reflected in the pre-clinical experimental design. Given the continued threat that zoonotic viruses pose to human populations, a treatment which utilises sialic acid is timely. Sialylated glycan-comprised receptors are ubiquitous in animals and humans and are conserved amongst different species, enabling efficient pan-species spillover of viruses [40]. Our results contribute to the evidence base for broad-spectrum therapies which can be applied across respiratory pathogens to counter existing and future threats to human health.
